# Scope downsizing enables circumferential endoscopic submucosal dissection in a narrowed, treatment-altered cervical esophagus

**DOI:** 10.1055/a-2978-3912

**Published:** 2026-10-28

**Authors:** Ludovico Alfarone, Francesco Minini, Antonio Capogreco, Davide Massimi, Cesare Hassan, Roberta Maselli, Alessandro Repici

**Affiliations:** 1 Digestive Endoscopy Unit 9268 IRCCS Humanitas Research HospitalRozzanoMilanItaly; 2 Department of Biomedical Sciences 437807 Humanitas UniversityPieve EmanueleMilanItaly; 3 Department of Biomedical Sciences 150525 Humanitas UniversityPieve EmanueleMilanItaly; 4 Digestive Endoscopic unit 9268 IRCCS Humanitas Research HospitalRozzanoMilanItaly

A 37-year-old woman with acute lymphoblastic leukemia treated with chemotherapy, total-body irradiation, and allogeneic stem-cell transplantation, and subsequent surgery for recurrent oropharyngeal squamous cell carcinoma was referred for a squamous neoplasm of the cervical esophagus. The Paris 0-IIb lesion extended from 19 to 22 cm from the incisors, involving half the circumference proximally and becoming fully circumferential centrally ( Video 1 ).

**Video 1** Scope downsizing with a 7.9-mm therapeutic slim gastroscope restored working space and maneuverability, enabling circumferential ESD of a superficial squamous neoplasm in a narrowed, treatment-altered cervical esophagus.


ESD is recommended for superficial esophageal squamous lesions, including selected circumferential cT1a-m1/m2 lesions 1​ . Here, the treatment-altered cervical esophagus was rigid and markedly narrowed. A conventional 9.3-mm gastroscope with a distal attachment could be advanced only with difficulty; wall compression and mucosal tearing during positioning prevented stable visualization and safe submucosal access.

The conventional gastroscope was therefore replaced with a 7.9-mm therapeutic slim gastroscope with a dedicated distal attachment. The smaller profile restored working space and rotational control. Therapeutic thin endoscopes have facilitated ESD in the cervical esophagus and near treatment-related strictures 2​ ​3​ , with data supporting use in stenosis or scarring 4​ ​5​ . This provided an intraprocedural rescue strategy when adequate working geometry could not be achieved.

After circumferential marking and submucosal injection, proximal and distal incisions were performed using a thin needle knife with high-pressure waterjet. Two opposing submucosal tunnels were created and connected by dissecting lateral bridges, allowing en bloc circumferential ESD in 70 minutes without adverse events. Diluted triamcinolone was injected into the post-ESD defect, followed by a prednisone taper for stricture prophylaxis.

Histology showed high-grade squamous intraepithelial neoplasia/squamous cell carcinoma in situ confined to the epithelium (m1), without lymphovascular invasion and with negative horizontal and vertical margins (R0). At endoscopic follow-up, no esophageal stricture was observed and the patient remained free of dysphagia.

Scope downsizing can restore the working space and maneuverability required for circumferential ESD in a narrowed, treatment-altered cervical esophagus.

Endoscopy_UCTN_Code_TTT_1AO_2AG_3AD

## Declaration of GenAI Use

Generative AI (ChatGPT, OpenAI) was used for translation and to improve the fluency of the English text.
